# Dysregulated transfer RNA-derived small RNAs as potential gastric cancer biomarkers

**DOI:** 10.3389/ebm.2024.10170

**Published:** 2024-12-13

**Authors:** Jie Yuan, Wenchao Gu, Tianxin Xu, Yan Zhang, Lei Shen, Jianliang Yan, Xi Guan, Haidan Chu, Ruoyu Yuan, Shaoqing Ju

**Affiliations:** ^1^ Department of Laboratory Medicine, Affiliated Hospital of Nantong University, Nantong University, Nantong, China; ^2^ Department of Special Laboratory Center, Affiliated Hospital of Nantong University, Nantong University, Nantong, China; ^3^ Department of General Surgery, Affiliated Hospital of Nantong University, Nantong University, Nantong, China; ^4^ Medical School of Nantong University, Nantong University, Nantong, China

**Keywords:** tsRNA, gastric cancer, tRF, diagnosis biomarker, ncRNA

## Abstract

Gastric cancer (GC) is the kind of carcinoma that has the highest rates of morbidity and death worldwide. In the early stages of GC, there is currently an absence of sensitive and specific biomarkers. The newly-discovered class of non-coding RNAs (ncRNAs) known as transfer RNA-derived small RNAs (tsRNAs) is highly expressed in bodily fluids and neoplastic cells. High-throughput sequencing was initially employed to identify differentially expressed tsRNAs in early GC patients, followed by validation in patient serum, GC tissues, and cell lines by quantitative real-time polymerase chain reaction (qRT-PCR). We identified dysregulated tsRNAs (the up-regulated tsRNAs included tRF-31-PNR8YP9LON4VD, tRF-30-MIF91SS2P4FI, and tRF-30-IK9NJ4S2I7L7, whereas the down-regulated tsRNAs included tRF-38-W6RM7KYUPRENRHD2, tRF-37-LBRY73W0K5KKOV2, tRF-36-JB59V3WD8YQ84VD, tRF-25-MBQ4NKKQBR, and tRF-36-0KFMNKYUHRF867D) in GC, and we verified that the serum of patients, GC cells and tissues both consistently expressed these tsRNAs. Additionally, GC patients’ serum had considerably greater expression levels of the three up-regulated tsRNAs than did healthy controls. Receiver operating characteristic (ROC) curve analysis demonstrated that the sensitivity and specificity of the three up-regulated tsRNAs were superior to those of CEA, CA199, and CA724 in the process of diagnosing GC, particularly in its early stages. This suggests that tsRNAs have great diagnostic efficacy and potential as new “liquid biopsy” biomarkers for the diagnosis of GC. Using bioinformatics software, we predicted that dysregulation of tsRNAs may be a potential regulatory mechanism for the development of GC.

## Impact statement

Gastric cancer (GC) is a malignancy characterized by a high global death rate and is deficient in sensitive diagnostic markers during its early stages. Recent studies have elucidated several functions of transfer RNA-derived small RNAs (tsRNAs) in many malignancies. The differential expression of tsRNAs in GC tissues and their biological importance were investigated in this study. Initially, we discovered that tsRNAs exhibited differential expression in patients with early gastric cancer by high-throughput sequencing. Moreover, the expression levels of tRF-31-PNR8YP9LON4VD, tRF-30-MIF91SS2P4FI, and tRF-30-IK9NJ4S2I7L7 in the serum of GC patients were significantly elevated compared to healthy controls, suggesting strong diagnostic potential, particularly in the early stages of gastric cancer. tsRNAs are considered to hold significant potential as innovative “liquid biopsy” biomarkers for the detection of GC.

## Introduction

Over one million new cases of gastric cancer (GC) are reported each year across the globe, with China being the location of forty percent of these occurrences. GC ranks among the top three malignancies in China in terms of morbidity and mortality [1, 2]. Furthermore, adenocarcinoma is the most common histopathological variant of GC due to the malignant transformation of gastric epithelial cells. Early-stage GC is indiscoverable because of its non-specific clinical symptoms, such as nausea. Upon diagnosis, over 70% of individuals exhibit locally advanced illness [3, 4]. Therefore, improving patient prognosis in GC through early diagnosis and treatment is of great clinical importance. While endoscopic screening is the primary method for early GC detection, early screening depends on the putative GC biomarkers’ ability to diagnose the disease. Haematological screening has several advantages, including convenience, cost-effectiveness and non-invasiveness [5]. At present, carcinoembryonic antigen (CEA), carbohydrate antigen 199 (CA199), and carbohydrate antigen 724 (CA724) are commonly used tumor biomarkers, but their specificity and sensitivity are limited [6]. Yu et al [7] showed that CEA had a sensitivity of approximately 13%–35% in diagnosing GC, whereas its specificity was approximately 65% only. Moreover, CA199 had a sensitivity of approximately 40% and specificity of approximately 70%. Consequently, there is an imperative necessity to develop novel clinical indicators for the early identification of GC.

Non-coding RNAs (ncRNAs) are the primary component of the human transcriptome [8]. Numerous research have investigated the involvement of ncRNAs families, including circular RNAs (circRNAs), long non-coding RNAs (lncRNAs), and microRNAs (miRNAs), in cancer formation and progression [9, 10]. Prior research indicated that ncRNAs may serve as biomarkers for diagnosing and prognosticating cancer patients [11]. Transfer RNA (tRNA) is a form of ncRNA that is universally expressed and conserved [12]. tRNA is crucial for maintaining normal homeostasis, cellular stress responses, stem cell formation, carcinogenesis, and the survival of cancer cells, comprising around 10% of total cellular RNA. The initial reports of tRNA-derived compounds originated in the late 1970s, when tRNA fragments were detected in cancer patients [13]. tsRNAs may be classified into two categories according to the cleavage sites of their source tRNA: tRNA-derived fragments (tRFs), which range from 14 to 36 nucleotides, and tRNA halves (tiRNAs), which range from 30 to 40 nucleotides [14, 15].

The importance of tsRNAs in cancer has recently attracted heightened attention due to their potential as innovative biomarkers [16]. Huang et al [17] shown that tDR-7816 expression promotes the growth early-stage breast cancer and was identified as a disease biomarker. Pekarsky et al [18] discovered that in lung cancer and chronic lymphocytic leukemia, ts-4521 and ts-3676 were down-regulated and shown anticancer properties. Shen et al [19] demonstrated that tRF-33-P4R8YP9LON4VDP promoted the proliferation and migration of GC cells while inhibiting cellular apoptosis, whereas Xu et al [20] discovered that tRF-Glu-TTC-027 impeded the progression of GC via modulating the MAPK signaling pathway. Our team’s prior research shown that three tsRNAs (tRF-29-RRJ89O9NF5JP, tRF-31-U5YKFN8DYDZDD, and tRF-23-Q99P9P9NDD) shown exceptional stability and specificity for GC patients’ dynamic monitoring [21–23].

Building on prior research, we further examined the therapeutic relevance of tsRNAs in GC. This work identified dysregulated tsRNAs using high-throughput sequencing. Through the validation of a significant number of serum and tissue samples, our aim is to identify tsRNAs that have biomarker roles. Furthermore, the specific mechanism of these tsRNAs in GC will be investigated in future experiments.

## Materials and methods

### Serum and tissue samples from clinical patients

This investigation followed the ethical standards set forth by the World Medical Association throughout the sample collection period from January 2021 to February 2024. Blood samples were collected from 60 patients diagnosed with primary hepatocellular cancer and 60 medical examiners at Affiliated Hospital of Nantong University. Three milliliters of blood were collected from each participant through venipuncture, and serum was isolated via centrifugation. Additionally, 36 pairs of GC tissues and the non-cancerous tissues that surrounded them were taken from our hospital’s operating room. All of the aforementioned patients received a pathology-confirmed diagnosis, gave their informed permission in accordance with ethical standards. Ethics committee approval was secured from the Affiliated Hospital of Nantong University (ethics review report number: 2018-L055).

### High-throughput sequencing

Total RNA isolation was performed with Trizol, followed by assessment of RNA quality and quantity. The Multi-sample library preparation kits was utilized to prepare a small RNA library using 1 μg total RNA from each sample. The library was sequenced, and the clean reads obtained were analyzed in comparison to MINTbase utilizing MINTmap software, leading to the generation of new tsRNA predictions for reads absent in MINTbase. The analysis of changes in tsRNAs across various groups was conducted using EdgeR software.

### Quantitative real-time polymerase chain reaction (qRT-PCR)

The qRT-PCR reaction was conducted utilizing a Bio-Rad CFX96 Real-Time PCR Detection System (Bio-Rad Laboratories, Inc., United States). U6 was utilized as an internal control, while RNA extracted from a pooled serum of 10 healthy donors acted as an external control. The sequences of the primers can be found in Supplementary Table S1. The 2^−ΔΔCT^ method was used to analyze the data using the formula: ΔΔCt = ΔCt_target_ (Ct_target_−Ct_reference_) - ΔCt_external control_ (Ct_external control_−Ct_reference_) during the reaction.

The amplification products of each tsRNA underwent verification through Sanger sequencing. The purified amplicons underwent sequencing with Applied Biosystems technology. Sequences underwent analysis with the ABI3730XL DNA Analyzer and were compared to tsRNA on a sequence-by-sequence basis.

### Cell culture

A set of human GC cell lines (MGC-803, AGS, MKN-45, SGC-7901, BGC-823) along with human gastric mucosal epithelial cell lines (GES-1) was sourced from the Chinese Academy of Sciences (Shanghai, China). All cells were cultured in RPMI 1640 medium (Corning, Manassas, VA, United States) enriched with 10% fetal bovine serum (Gibco, Waltham, MA, United States) and 1% penicillin and streptomycin (HyClone, Logan, UT, United States) under standard conditions of 37°C and 5% CO_2_ in a humidified incubator.

### Total RNA extraction and RNA reverse transcription

Total RNA was extracted from serum samples of GC patients utilizing a total RNA purification and isolation kit (BioTeke, Wuxi, Jiangsu, China). In contrast, total RNA from tissue and cell samples was obtained using TRIzol reagent (Invitrogen, Carlsbad, CA, United States). The process of reverse transcription of total RNA to cDNA was executed with a revert aid RT reverse transcription kit (Thermo Fisher Scientific, Waltham, MA, United States). This was carried out in a 10 µL reaction volume, incubated at 42°C for 60 min, and subsequently inactivated at 70°C for 5 min. Prior to the reaction, quantification was necessary.

### Bioinformatics analysis

The nomenclature and structures of tsRNAs: MINTbase version 2.0,^1^ UCSC Genome Browser database,^2^ and Transfer RNA Database.^3^

Identification of tsRNAs and Target Genes: Miranda,^4^ and Pictar.^5^

Screening for Differentially Expressed Genes: Kyoto Encyclopedia of Genes and Genomes (KEGG),^6^ Gene Ontology enrichment analysis^7^ and Reactome Pathway Database.^8^

### Statistical analysis

The analysis of high-throughput sequencing results was conducted utilizing the R package edgeR, with significance determined at *p* < 0.05 and |Log_2_FC|>2. The analysis of data was conducted using SPSS Statistics Version 20.0 (IBM SPSS Statistics, Chicago, IL, United States) and GraphPad Prism 9.0 (GraphPad Software, San Jose, California, United States). Prior to conducting the analysis, the normality of the data was evaluated through normality and lognormality tests, which confirmed that all data adhered to a normal distribution. Group differences were assessed through the application of t-tests. The ROC curve and the area under the curve (AUC) were utilized to assess the diagnostic efficacy of tsRNAs in GC. Prior to plotting the ROC curve, binomial logistic regression was conducted. Cox’s proportional hazards model was utilized for the multivariate analysis. The risk ratio along with its 95% confidence interval (CI) was documented for each marker. The determination of tsRNA cut-off values utilized the Youden index, whereas the cut-off values for CEA, CA199, and CA724 were established based on the reference range from the Affiliated Hospital of Nantong University, specifically 5 ng/mL, 37 U/mL, and 10 U/mL, respectively. Independent experiments were conducted a minimum of three times, and statistical significance was established when the *P*-value was less than 0.05.

## Results

### Expression profiles of tsRNAs in GC and preliminary validation

**FIGURE 1:**
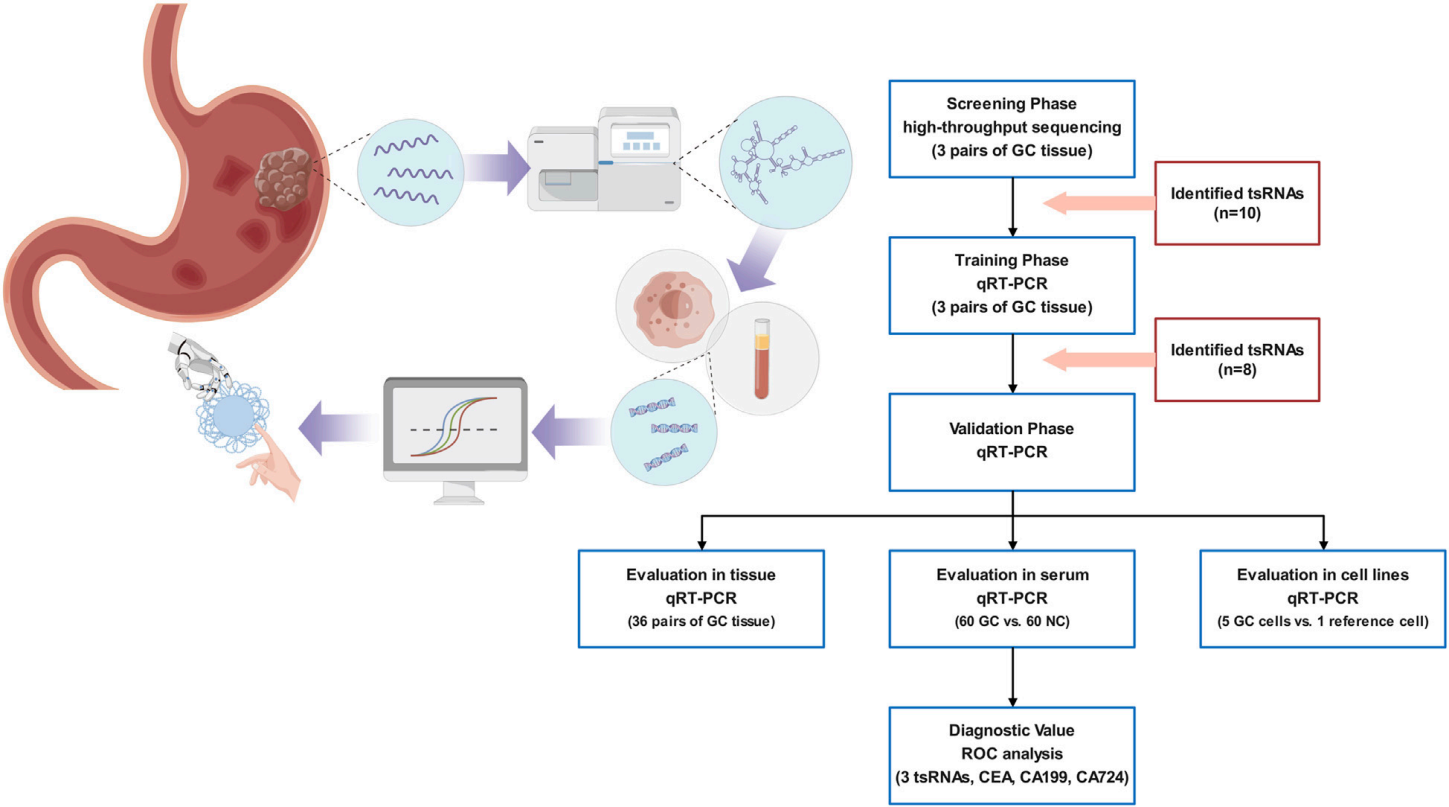
An illustration of the research methodology.

The deregulation of ncRNA expression is closely associated with the initiation and advancement of cancers. Studies have focused on ncRNAs as potential diagnostic biomarkers because of their tissue specificity and convenient and stable detection in human tissues and body fluids. tsRNAs have been shown to circulate in a very stable, cell-free state inside the serum. The study design is presented in Figure 1. We initially utilized a high-throughput sequencing technique to identify the differentially expressed tsRNAs in GC tissues and adjacent non-cancerous specimens. The identified tsRNAs with *p* < 0.05 and |Log_2_FC|>2 were differentially expressed.

**FIGURE 2:**
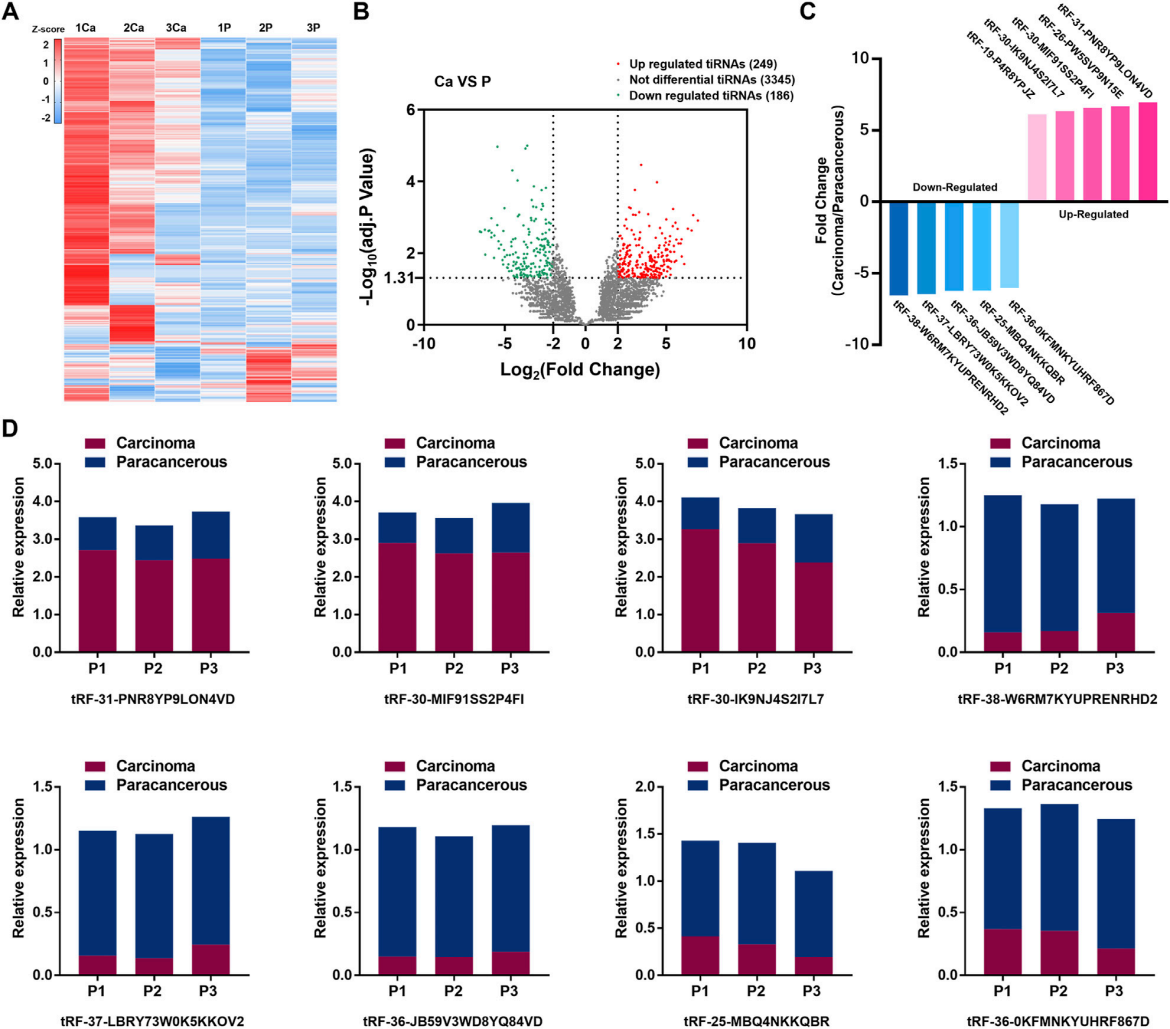
Expression profiles of tsRNAs in GC and preliminary validation. **(A)** High-throughput sequencing performed on three pairs of TNM I/II stage GC tissues and adjacent nontumor tissue, the expression heatmap of high-throughput sequencing. **(B)** Volcano plot of tsRNAs distribution. **(C)** 10 candidates selected from the top five up-regulated tsRNAs and top five down-regulated tsRNAs. **(D)** qRT-PCR validated 10 candidates and 8 tsRNAs conformed.

Statistical results showed 435 tsRNAs were dysregulated (249 were up-regulated, and 186 were down-regulated). The expression heatmap and volcano plot are presented in Figures 2A, B. Ten candidates were selected from the top five up-regulated and top five down-regulated tsRNAs, based on the average expression level and the uniformity of fluorescence signals within each group (Figure 2C; Supplementary Table S2). To confirm the results of RNA sequencing, we employed the same three pairs of GC tissues for qRT-PCR. The results demonstrated that eight tsRNAs (the up-regulated tsRNAs include: tRF-31-PNR8YP9LON4VD, tRF-30-MIF91SS2P4FI, tRF-30-IK9NJ4S2I7L7, and the down-regulated tsRNAs include: tRF-38-W6RM7KYUPRENRHD2, tRF-37-LBRY73W0K5KKOV2, tRF-36-JB59V3WD8YQ84VD, tRF-25-MBQ4NKKQBR, tRF-36-0KFMNKYUHRF867D, tsRNAs named followed version 2.0 of MINTbase) expression levels were consistent with the high-throughput sequencing (Figure 2D).

### Expression of tsRNAs in GC tissues and cell lines

**FIGURE 3:**
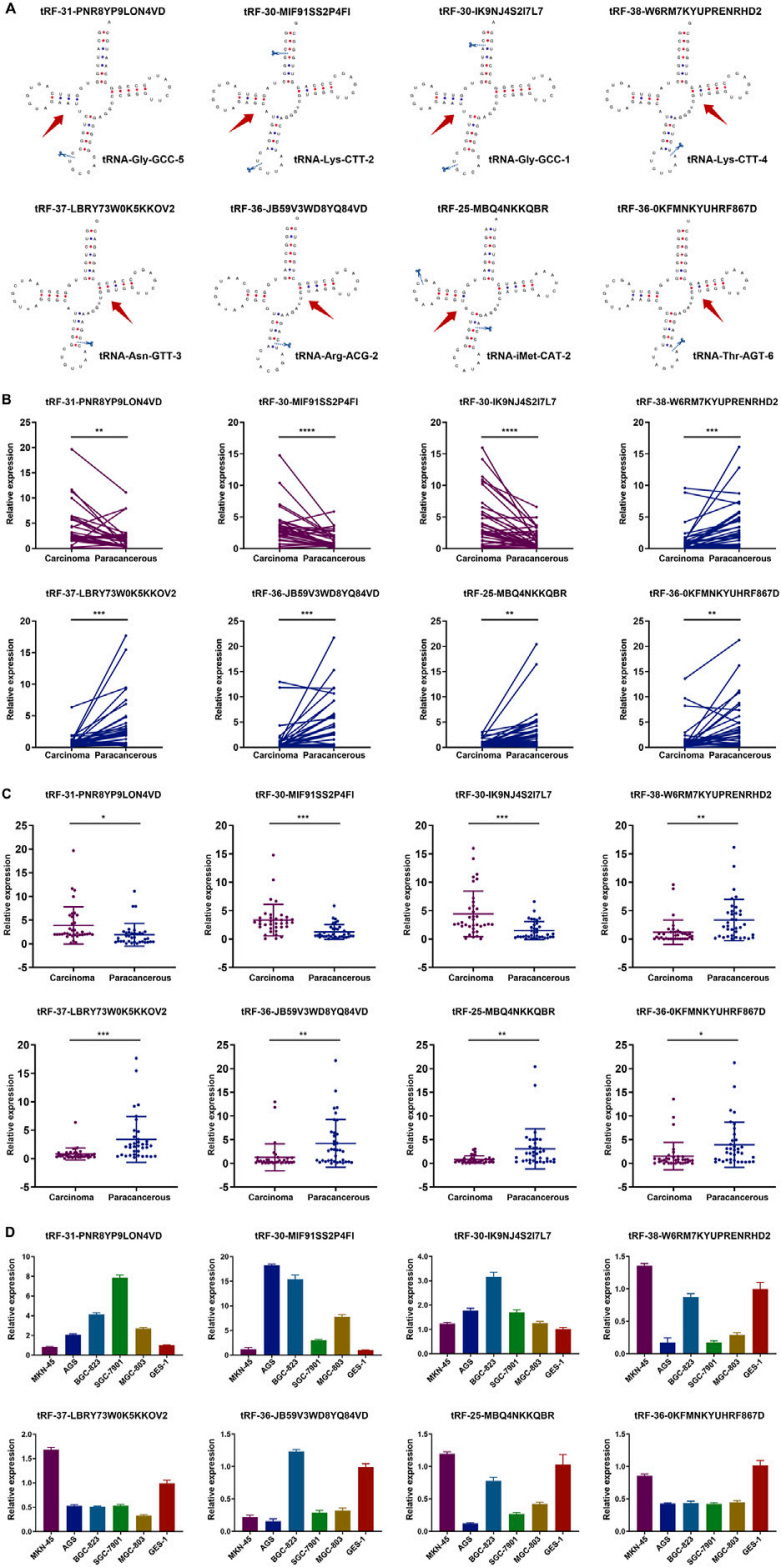
Structure of tsRNAs and expression in GC tissues and cell lines. **(A)** The structure and cleavage site of the eight tsRNAs. **(B)** Expression of the eight tsRNAs in 36 pairs of tissue samples from GC patients. Statistics by paired t-tests. **(C)** Expression of the eight tsRNAs in 36 pairs of tissue samples from GC patients. Statistics by unpaired t-tests. **(D)** Expression of the eight tsRNAs in five human GC cell lines (MGC-803, AGS, MKN-45, SGC-7901, BGC-823) and the human gastric epithelial cell line (GES-1). All the values are as mean ± SD. ****P < 0.0001, ***P < 0.001, **P < 0.01, *P < 0.05.

The structure and cleavage site of the eight tsRNAs were visualized in Figure 3A. To further validate the eight tsRNAs expression levels, we obtained 36 pairs of tissue samples from GC patients and assessed the expression levels of the eight tsRNAs. Results showed that the three up-regulated tsRNAs expressed higher levels in carcinomas than paracancerous specimens, and the five down-regulated tsRNAs were lowly expressed (Figure 3B). The results of unpaired comparisons were consistent with the results of paired comparisons within 36 pairs of tissue samples (Figure 3C). Simultaneously, we observed the expression levels of eight tsRNAs across several GC cell lines and discovered that the three up-regulated tsRNAs were markedly elevated in GC cells relative to GES-1 cell line, whereas the five down-regulated tsRNAs exhibited the inverse pattern (Figure 3D). This section assessed the expression of eight tsRNAs in GC tissue samples and cell lines, revealing that these tsRNAs were dysregulated in GC.

### Diagnostic efficiency of tsRNAs in serum of GC patients

**FIGURE 4:**
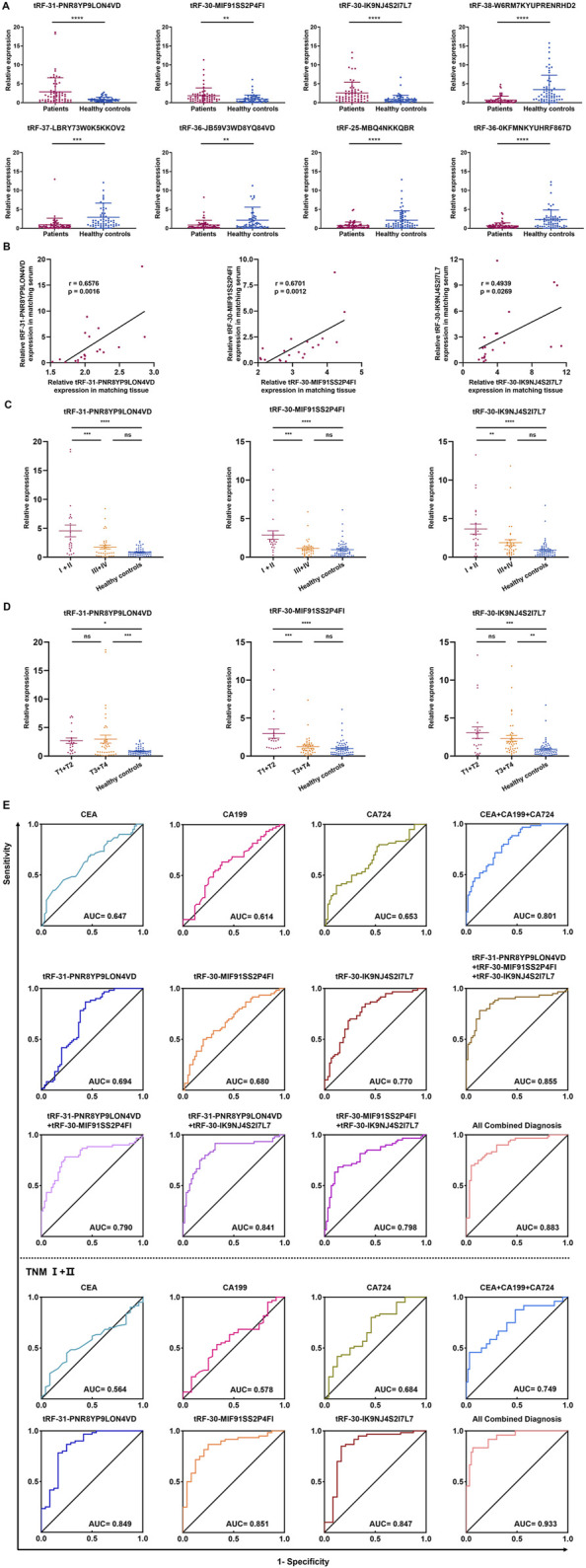
Expression and diagnostic efficiency of tsRNAs in serum of GC patients. **(A)** The eight tsRNAs were expressed in 60 GC patient and 60 health evaluation serum samples. **(B)** The expression levels of three up-regulated tsRNAs (tRF-31-PNR8YP9LON4VD, tRF-30-MIF91SS2P4FI, and tRF-30-IK9NJ4S2I7L7) were analyzed using Pearson correlation in 20 GC tissues and serum samples from patients with matching diagnoses. **(C)** The expression levels of tRF-31-PNR8YP9LON4VD, tRF-30-MIF91SS2P4FI, and tRF-30-IK9NJ4S2I7L7 in serum of TNM I/II stage GC patients, TNM III/IV stage GC patients, and health examination subjects. **(D)** The expression levels of tRF-31-PNR8YP9LON4VD, tRF-30-MIF91SS2P4FI, and tRF-30-IK9NJ4S2I7L7 in different stages of the depth of tumor invasion and health examination subjects. **(E)** ROC curves for GC diagnosis or TNM I+II stage using serum tRF-31-PNR8YP9LON4VD, tRF-30-MIF91SS2P4FI, tRF-30-IK9NJ4S2I7L7, CEA, CA199, CA724 and combined diagnosis between GC patients and healthy controls. All the values are as mean ± SD. ****P < 0.0001, ***P < 0.001, **P < 0.01, *P < 0.05.

The collection of peripheral blood samples from patients for testing is the most convenient diagnostic method; hence, we sought to determine if these tsRNAs were differentially expressed in the serum of 60 GC patients compared to 60 healthy controls. The serum concentration of tRF-31-PNR8YP9LON4VD, tRF-30-MIF91SS2P4FI, and tRF-30-IK9NJ4S2I7L7 showed a statistically significant increase in GC patients, whereas tRF-38-W6RM7KYUPRENRHD2, tRF-37-LBRY73W0K5KKOV2, tRF-36-JB59V3WD8YQ84VD, tRF-25-MBQ4NKKQBR, and tRF-36-0KFMNKYUHRF867D showed an opposite trend (Figure 4A). The expression levels of tRF-31-PNR8YP9LON4VD, tRF-30-MIF91SS2P4FI, and tRF-30-IK9NJ4S2I7L7 in tissues were correlated with the expression levels in the same patient’s serum (Figure 4B).

For each tsRNA group, we split 60 GC patients in half, with 30 patients in the high expression group and 30 in the low expression group, according to the median serum expression of tRF-31-PNR8YP9LON4VD, tRF-30-MIF91SS2P4FI, and tRF-30-IK9NJ4S2I7L7, respectively. Clinicopathological parameters were evaluated the correlations with the three up-regulated tsRNAs expression levels in serum using the χ^2^ test. The results demonstrated a strong correlation between gender (*p* = 0.0321), differentiation grade (*p* = 0.0062), and TNM stage (*p* = 0.035) and tRF-31-PNR8YP9LON4VD expression. There was a significant correlation between the expression of tRF-30-MIF91SS2P4FI and age (*p* = 0.0242), TNM stage (*p* = <0.0001), tumor depth (*p* = 0.0149), and lymph node metastasis (*p* = 0.0242). A statistically significant connection (*p* = 0.0016) was found between the expression of tRF-30-IK9NJ4S2I7L7 and TNM stage (Table 1). The elevated expression of tsRNAs may serve as a possible indicator for predicting tumor malignant development. Subsequently, we categorized the substantially varied clinicopathological features into groups to further evaluate the disparities in tsRNA expression levels within each group. The results demonstrated that the expression of three upregulated tsRNAs increased with the early TNM stages (I/II) and T1/T2 tumor depth of gastric cancer (Figures 4C, D).

**TABLE 1:** Clinicopathological parameters of gastric cancer patients.

Characteristics	Screening/training phase	Validation phase			tRF-31-PNR8YP9LON4VD			tRF-30-MIF91SS2P4FI			tRF-30-IK9NJ4S2I7L7		
	GC tissue samples (%)	GC tissue samples (%)	NC serum samples (%)	GC serum samples (%)	High	Low	*p* Value	High	Low	*p* Value	High	Low	*p* Value
Number	3	36	60	60	30	30		30	30		30	30	
Gender													
Male	2 (66.7)	23 (63.9)	30 (50.0)	38 (63.3)	23	15	*0.0321	21	17	0.2839	21	17	0.2839
Female	1 (33.3)	13 (36.1)	30 (50.0)	22 (36.7)	7	15		9	13		9	13	
Age (Years)													
< 60	1 (33.3)	11 (30.6)	26 (43.3)	18 (30.0)	6	12	0.091	13	5	*0.0242	7	11	0.2598
≥ 60	2 (66.7)	25 (69.4)	34 (56.7)	42 (70.0)	24	18		17	25		23	19	
Differentiation grade													
Well-moderate	2 (66.7)	12 (33.3)		20 (33.3)	15	5	**0.0062	12	8	0.2733	13	7	0.1003
Poor-undifferentiation	1 (33.3)	24 (66.7)		40 (66.7)	15	25		18	22		17	23	
TNM stage													
I + II	3 (100)	20 (55.6)		24 (40.0)	16	8	*0.0350	20	4	****<0.0001	18	6	**0.0016
III + IV	0 (0)	16 (44.4)		36 (60.0)	14	22		10	26		12	24	
Tumor depth													
T1-T2	3 (100)	16 (44.4)		21 (35.0)	12	9	0.4168	15	6	*0.0149	13	8	0.176
T3-T4	0 (0)	20 (55.6)		39 (65.0)	18	21		15	24		17	22	
Lymph node metastasis													
Negative	2 (66.7)	14 (38.9)		18 (30.0)	8	10	0.5731	5	13	*0.0242	11	7	0.2598
Positive	1 (33.3)	22 (61.1)		42 (70.0)	22	20		25	17		19	23	
Distant metastasis													
Absence	3 (100)	28 (77.8)		50 (83.3)	26	24	0.4884	23	27	0.1659	26	24	0.4884
Presence	0 (0)	8 (22.2)		10 (16.7)	4	6		7	3		4	6	

We further assessed the diagnostic effectiveness of three highly expressed tsRNAs for GC based on their expression levels in patients with GC using ROC analysis. The diagnostic AUC values of tRF-31-PNR8YP9LON4VD, tRF-30-MIF91SS2P4FI, and tRF-30-IK9NJ4S2I7L7 were 0.808 (95% confidence interval, 0.694–0.924), 0.774 (95% CI, 0.657–0.892), and 0.843 (95% CI, 0.747–0.940), respectively (Figure 4E). We compared the diagnostic efficiency of the three up-regulated tsRNAs with GC clinical biomarkers CEA, CA199, and CA724. ROC analysis indicated that the three tsRNAs had better diagnostic efficacy than CEA, CA199, and CA724. AUC increased to 0.956 (95% CI, 0.906–1) when all biomarkers were combined for diagnosis. Additionally, we analysed the diagnostic efficacy of the three tsRNAs in TNM Ⅰ/Ⅱ stage of GC based on the clinicopathological parameters of the patients. We discovered that their diagnostic value increases even further in the early stages of GC. The diagnostic AUC values of tRF-31-PNR8YP9LON4VD, tRF-30-MIF91SS2P4FI, and tRF-30-IK9NJ4S2I7L7 were 0.849, 0.851 and 0.847 (Figure 4E). In contrast, CEA and CA199 have limited diagnostic value (AUC values of 0.564 and 0.578) in the early stages of GC.

### Predicted targets and functions of tsRNAs in GC

**FIGURE 5:**
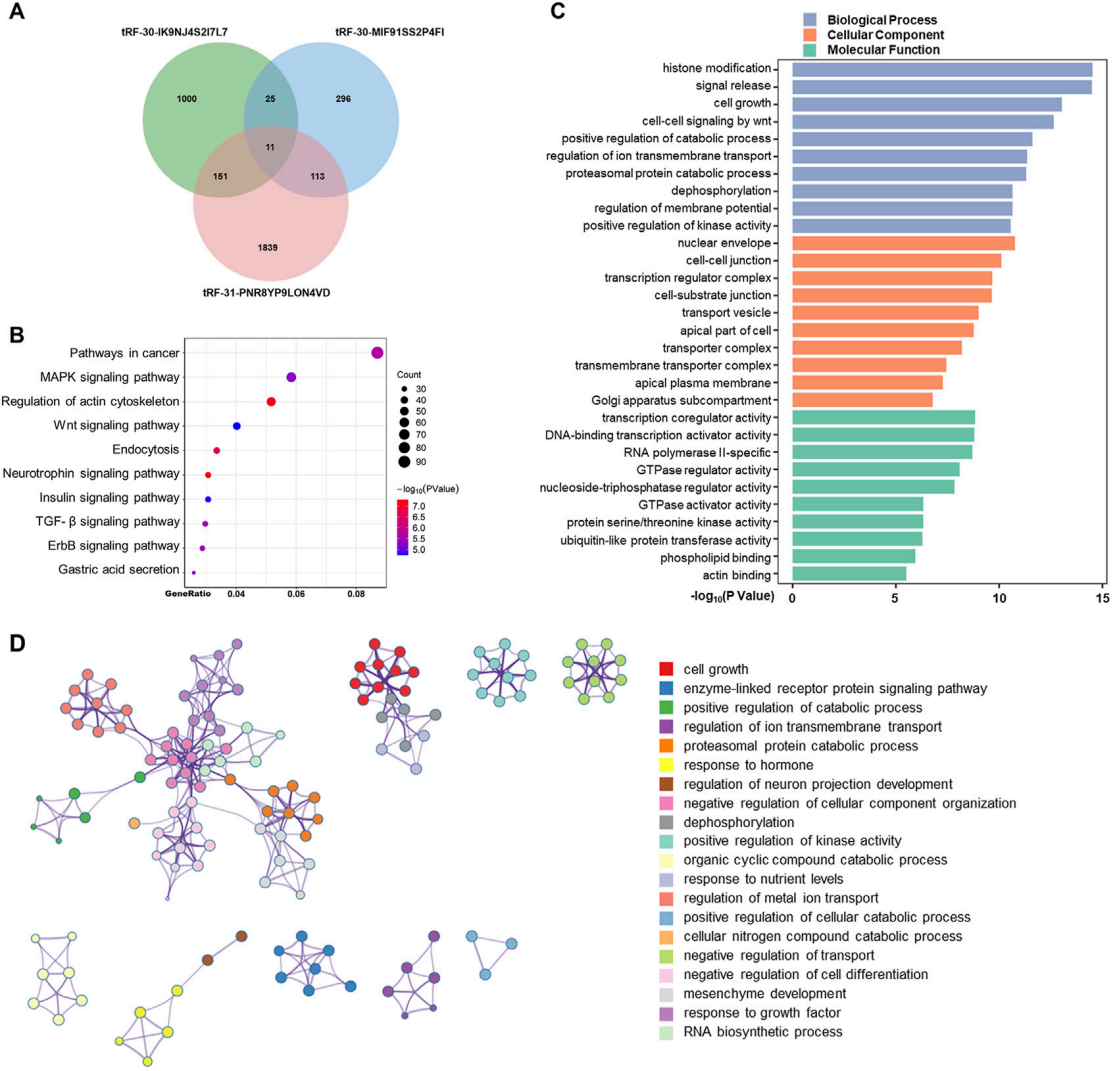
Predicted targets and functions of tsRNAs in GC. **(A)** Using miRanda and PicTar databases, predicted downstream target genes of tRF-31-PNR8YP9LON4VD, tRF-30-MIF91SS2P4FI, and tRF-30-IK9NJ4S2I7L Venning diagrams examined the three tsRNAs’ target genes’ overlap. **(B)** Bubble graphic showing KEGG analysis of pathway-enriched target genes. **(C)** GO analysis of biological processes, cellular components, and molecular functions-enriched target genes. **(D)** Enhanced terminology network. We grouped terms with a *p*-value < 0.01, a minimum count of 3, and an enrichment factor >1.5 into clusters based on membership similarities. Kappa values of 4 were utilized for hierarchical clustering of enriched phrases, with sub-trees with similarity larger than 0.3 identified as clusters.

Consequently, we aimed to further examine and forecast the involvement of these tsRNAs in the advancement of gastric cancer. We predicted downstream target genes of tRF-31-PNR8YP9LON4VD, tRF-30-MIF91SS2P4FI, and tRF-30-IK9NJ4S2I7L7 using miRanda and PicTar databases. The prediction results of the two databases were intersected, and the results showed 1,187 target genes that were most likely bound to tRF-30-IK9NJ4S2I7L7, 445 to tRF-30-MIF91SS2P4FI, and 2,114 to tRF-31-PNR8YP9LON4VD (Figure 5A). Integrating all 3,435 predicted target genes, the KEGG signaling network was shown to be considerably enriched in pathways related to cancer, the MAPK signaling pathway, and the control of the actin cytoskeleton, according to an enrichment analysis (Figure 5B). Gene Ontology functional enrichment study suggested that the up-regulated tsRNAs of GC may play a significant role in histone modification, signal release, and cell growth in biological processes (Figure 5C). The phrase having the highest statistical significance inside a cluster was selected to represent that cluster. The enrichment analysis is depicted in Figure 5D. It is evident that certain genes play a role in multiple functions. Nonetheless, the processes behind tsRNA expression in the control of biological activity in GC cells require additional investigation.

## Discussion

GC is among the most aggressive malignancies globally. Notwithstanding advancements in surgical procedures, radiation, and chemotherapy, the incidence and fatality rates of GC persist as the fifth and fourth highest globally, respectively [24]. Early-stage GC frequently presents with vague symptoms, and 80% of patients are discovered at advanced stages, so forfeiting the chance for surgical resection [25]. Currently, there are no biomarkers for GC that fulfill the necessary requirements; thus, efforts are underway to identify novel biomarkers with enhanced sensitivity and specificity to facilitate early diagnosis of GC [26].

There is mounting evidence from high-throughput sequencing studies linking dysregulated ncRNA expression to tumor formation and progression [11]. ncRNAs were increasingly recognized as a diagnostic tumor marker because of its remarkable tissue specificity and ability to be detected conveniently and robustly in human tissues and body fluids [27–29]. In 1979, tsRNA, a newly discovered family of ncRNA, was first found in patients with cancer, indicating that tsRNAs participate in the gene expression regulation in these patients [13]. Through their interactions with proteins and mRNAs, tsRNAs regulate gene expression, epigenetic changes, and the cell cycle, among other biological processes [30, 31]. A number of studies have identified dysregulated tsRNAs in various tumor types using high-throughput sequencing methods [32–34]. Investigating the particular pathways of dysregulated tsRNAs is crucial for the early detection and treatment of GC.

A total of three sets of tissues were sequenced using high-throughput technology in order to identify tsRNAs that were differently expressed. One set of tissues were from early-stage GC, while the other set came from nearby non-tumor tissues. After that, we used qRT-PCR to confirm in GC tissues and cell lines the top five tsRNAs that were up-regulated and the top five tsRNAs that were down-regulated. Three up-regulated and five down-regulated tsRNAs were consistent with high-throughput sequencing results. Nevertheless, it is expected that down-regulated tsRNAs will be identified in significant quantities in healthy populations due to their probable involvement in cancer development. Nevertheless, we acknowledge the challenge of determining the minimum threshold of the reference value for the down-regulated tsRNAs. Therefore, we intend to concentrate our future investigations on the oncogenic functions of these five tsRNAs in relation to GC. For the present biomarker investigation, our main emphasis was on the three tsRNAs that demonstrated high expression levels.

We collected serum samples from 60 individuals recently diagnosed with GC and 60 healthy controls to determine the expression levels of these tsRNAs in the serum. All three of the up-regulated tsRNAs were highly expressed in GC patients, according to the data.

A large body of research suggests that tsRNAs may serve as biomarkers for GC due to their distinct expression patterns in the blood of people with the disease. Many of these studies examined the expression level of tsRNAs using receiver operating characteristic (ROC) but did not assess the diagnostic effectiveness with widely used diagnostic markers of gastric cancer, such as CEA, CA199, and CA724. In a study conducted by Mao et al. [35], the researchers investigated how well tsRNAs might detect GC in its early stages. The possible advantages for early detection of GC could not be investigated due to the study’s tiny sample size. Consequently, we set out to learn more about how well GC may be detected in its early stages. ROC analysis showed that tRF-31-PNR8YP9LON4VD, tRF-30-MIF91SS2P4FI and tRF-30-IK9NJ4S2I7L7 had higher sensitivity and specificity and were superior to the conventional markers (CEA, CA199, and CA724) in the differential diagnosis of GC, particularly in the early stages of the disease. The combination of tRF-31-PNR8YP9LON4VD, tRF-30-MIF91SS2P4FI, and tRF-30-IK9NJ4S2I7L7 with CEA, CA199, and CA724 has enhanced diagnostic efficacy and significant clinical promise.

Similar to microRNAs, tsRNAs have been shown to regulate certain genes or signaling cascades and may act as tumor suppressors or oncogenes. Shen et al [19] observed that tRF-33-P4R8YP9LON4VDP inhibited apoptosis and increased GC cell migration and proliferation, suggesting that this tsRNA represents a viable target for targeted treatment. Xu et al [20] discovered that tRF-Glu-TTC-027 inhibited GC cell growth via regulating the MAPK signaling cascade. In some malignancies, such as breast cancer, the RUNT-related transcription factor can mitigate the effects of ts-112, which cause tumor cells to proliferate at an enhanced pace [36]. In breast cancer, tsRNA-26576 not only promotes tumor cell proliferation but also the invasion and migration [37]. Therefore, we predicted the potential downstream genes regulated by tRF-31-PNR8YP9LON4VD, tRF-30-MIF91SS2P4FI, and tRF-30-IK9NJ4S2I7L7 through miRanda and PicTar databases. There is speculation that GC-enriched tsRNAs may play a role in certain signaling pathways associated with cancer.

Our preliminary study identified three up-regulated and five down-regulated tsRNAs through high-throughput sequencing. Further analysis of the highly expressed tsRNAs revealed a correlation between their serum expression levels and GC TNM stage. ROC curve analysis suggested that these tsRNAs have superior diagnostic value for early-stage GC compared to traditional biomarkers. However, the study has limitations. The samples provided are inadequate, and the clinical use of these biomarkers has not yet been established. Additionally, it is necessary to monitor changes in tsRNA expression levels during disease progression and their potential as prognostic indicators in GC patients. Further investigation into the function of tsRNAs in GC cells and the methods by which they contribute to GC will be carried out in the near future.

## Conclusion

Using high-throughput sequencing, we discovered dysregulated tsRNAs in GC tissues. The expression levels of tRF-31-PNR8YP9LON4VD, tRF-30-MIF91SS2P4FI, and tRF-30-IK9NJ4S2I7L7 in the serum of patients with GC were considerably elevated compared to healthy controls, demonstrating substantial diagnostic effectiveness. Therefore, tsRNAs have great potential becoming new “liquid biopsy” biomarkers for the diagnosis of GC. Through bioinformatics software, we predicted that dysregulated tsRNAs have potential regulatory mechanisms for GC development. Our next research will focus on examining the functional role of tsRNAs in GC, and our results lay the theoretical groundwork for that investigation. In conclusion, emerging ncRNAs linked to GC indicate that tsRNAs may serve as biomarkers and predictors of worse prognosis. Dysregulated tsRNAs can be potential therapeutic targets for GC.

## Data Availability

The datasets presented in this study can be found in online repositories. The names of the repository/repositories and accession number(s) can be found in the article/Supplementary Material.

## Footnotes

1
https://cm.jefferson.edu/MINTbase/

2
https://genome.ucsc.edu/

3
http://trna.bioinf.unileipzig.de/

4
http://www.microrna.org/

5
https://pictar.mdc-berlin.de/

6
https://www.genome.jp/kegg/

7
https://geneontology.org/docs/go-enrichment-analysis/

8
https://reactome.org/
