# Supplementary material for: Dysregulated transfer RNA-derived small RNAs as potential gastric cancer biomarkers
Source: Exp Biol Med (Maywood). 2024 Dec 13;249:10170. doi: 10.3389/ebm.2024.10170 (PMC11673218; doi:10.3389/ebm.2024.10170)
Supplement: Supplementary file 1 [file Table1.DOCX]

Supplementary Table 1. The primer sequences of tsRNAs

| tsRNA label | sequence (5'-3') |
| --- | --- |
| tRF-38-W6RM7KYUPRENRHD2 | UCUCAGGGUCGUGGGUUCGAGCCCCACGUUGGGCGCCA |
| RT primer | GTCGTATCCAGTGCAGGGTCCGAGGTATTCGCACTGGATACGACTGGCGC |
| Forward primer | TCGAGCCCCACGTTGG |
| Reverse primer | AGTGCAGGGTCCGAGGTATT |
|  |  |
| tRF-37-LBRY73W0K5KKOV2 | CGAAAGGUUGGUGGUUCGAGCCCACCCAGGGACGCCA |
| RT primer | GTCGTATCCAGTGCAGGGTCCGAGGTATTCGCACTGGATACGACTGGCGT |
| Forward primer | TCGAGCCCACCCAGGG |
| Reverse primer | AGTGCAGGGTCCGAGGTATT |
|  |  |
| tRF-36-JB59V3WD8YQ84VD | CAGAAGAUUCUAGGUUCGACUCCUGGCUGGCUCGCC |
| RT primer | GTCGTATCCAGTGCAGGGTCCGAGGTATTCGCACTGGATACGACGGCGAG |
| Forward primer | GGTTCGACTCCTGGCTGG |
| Reverse primer | AGTGCAGGGTCCGAGGTATT |
|  |  |
| tRF-25-MBQ4NKKQBR | CGGAAGCGUGCUGGGCCCAUAACCC |
| RT primer | GTCGTATCCAGTGCAGGGTCCGAGGTATTCGCACTGGATACGACGGGTTA |
| Forward primer | AAGCGTGCTGGGCCCA |
| Reverse primer | AGTGCAGGGTCCGAGGTATT |
|  |  |
| tRF-36-0KFMNKYUHRF867D | ACAGGAGAUCCUGGGUUCGAAUCCCAGCGGGGCCUC |
| RT primer | GTCGTATCCAGTGCAGGGTCCGAGGTATTCGCACTGGATACGACGAGGCC |
| Forward primer | GGTTCGAATCCCAGCGG |
| Reverse primer | AGTGCAGGGTCCGAGGTATT |
|  |  |
| tRF-31-PNR8YP9LON4VD | GCAUUGGUGGUUCAGUGGUAGAAUUCUCGCC |
| RT primer | GTCGTATCCAGTGCAGGGTCCGAGGTATTCGCACTGGATACGACGGCGAG |
| Forward primer | CATTGGTGGTTCAGTGGTAGAATT |
| Reverse primer | AGTGCAGGGTCCGAGGTATT |
|  |  |
| tRF-26-PW5SVP9N15E | GCCGUGAUCGUAUAGUGGUUAGUACU |
| RT primer | GTCGTATCCAGTGCAGGGTCCGAGGTATTCGCACTGGATACGACAGTACT |
| Forward primer | GGCCGTGATCGTATAGTGGTT |
| Reverse primer | AGTGCAGGGTCCGAGGTATT |
|  |  |
| tRF-30-MIF91SS2P4FI | CGGCUAGCUCAGUCGGUAGAGCAUGAGACU |
| RT primer | GTCGTATCCAGTGCAGGGTCCGAGGTATTCGCACTGGATACGACAGTCTC |
| Forward primer | GCTAGCTCAGTCGGTAGAGCAT |
| Reverse primer | AGTGCAGGGTCCGAGGTATT |
|  |  |
| tRF-30-IK9NJ4S2I7L7 | AUGGGUGGUUCAGUGGUAGAAUUCUCGCCU |
| RT primer | GTCGTATCCAGTGCAGGGTCCGAGGTATTCGCACTGGATACGACAGGCGA |
| Forward primer | TGGGTGGTTCAGTGGTAGAATTC |
| Reverse primer | AGTGCAGGGTCCGAGGTATT |
|  |  |
| tRF-19-P4R8YPJZ | GCAUGGGUGGUUCAGUGGU |
| RT primer | GTCGTATCCAGTGCAGGGTCCGAGGTATTCGCACTGGATACGACACCACT |
| Forward primer | CGCGGCATGGGTGGTTC |
| Reverse primer | AGTGCAGGGTCCGAGGTATT |
