# Supplementary material for: Dysregulated transfer RNA-derived small RNAs as potential gastric cancer biomarkers
Source: Exp Biol Med (Maywood). 2024 Dec 13;249:10170. doi: 10.3389/ebm.2024.10170 (PMC11673218; doi:10.3389/ebm.2024.10170)
Supplement: Supplementary file 2 [file Table2.docx]

**Supplementary Table 2. The top 10 candidates of high-throughput sequencing technique in three pairs of GC tissues and matched paracancerous specimens**

| gene_symbol | log_2_FC |
| --- | --- |
| tRF-38-W6RM7KYUPRENRHD2 | -6.54055 |
| tRF-37-LBRY73W0K5KKOV2 | -6.46645 |
| tRF-36-JB59V3WD8YQ84VD | -6.22726 |
| tRF-25-MBQ4NKKQBR | -6.20726 |
| tRF-36-0KFMNKYUHRF867D | -6.00658 |
| tRF-19-P4R8YPJZ | 6.11902 |
| tRF-30-IK9NJ4S2I7L7 | 6.34075 |
| tRF-30-MIF91SS2P4FI | 6.56823 |
| tRF-26-PW5SVP9N15E | 6.67416 |
| tRF-31-PNR8YP9LON4VD | 6.95758 |
